# The impact of translation modality on user experience: an eye-tracking study of the Microsoft Word user interface

**DOI:** 10.1007/s10590-021-09267-z

**Published:** 2021-06-22

**Authors:** Ana Guerberof Arenas, Joss Moorkens, Sharon O’Brien

**Affiliations:** 1 Centre for Translation Studies, University of Surrey, Guildford, UK; 2 Centre for Language and Cognition, University of Groningen, Groningen, The Netherlands; 3 School of Applied Language and Intercultural Studies, Dublin City University, Dublin, Ireland

**Keywords:** Usability, Machine translation, Human translation, Eye-tracking, Human–computer interaction

## Abstract

This paper presents results of the effect of different translation modalities on users when working with the Microsoft Word user interface. An experimental study was set up with 84 Japanese, German, Spanish, and English native speakers working with Microsoft Word in three modalities: the published translated version, a machine translated (MT) version (with unedited MT strings incorporated into the MS Word interface) and the published English version. An eye-tracker measured the cognitive load and usability according to the ISO/TR 16982 guidelines: i.e., effectiveness, efficiency, and satisfaction followed by retrospective think-aloud protocol. The results show that the users’ effectiveness (number of tasks completed) does not significantly differ due to the translation modality. However, their efficiency (time for task completion) and self-reported satisfaction are significantly higher when working with the released product as opposed to the unedited MT version, especially when participants are less experienced. The eye-tracking results show that users experience a higher cognitive load when working with MT and with the human-translated versions as opposed to the English original. The results suggest that language and translation modality play a significant role in the usability of software products whether users complete the given tasks or not and even if they are unaware that MT was used to translate the interface.

## Introduction

The software and localization industries face long-term business challenges. According to Statista,^1^ global software market revenue is projected to be 466.8 billion US dollars for 2019, rising to 507.2 billion US dollars for 2021 and according to Nimdzi’s Software Localization Report,^2^ the software sector has a growth rate of 8.3% and is the fastest-growing sector in the global IT industry. Consequently, there is an increase in the volume of software to localize and this software needs to run on several platforms and be delivered to the user via a rapid, agile development cycle, with daily, weekly, and quarterly updates and releases. The market size of the global language services industry is projected to reach 51.8 billion US dollars in 2021.^3^ In parallel, there are continuous advances in machine translation (MT) technology (Vaswani et al. 2017), and full implementations of MT solutions in the translation workflow.^4^ It is, therefore, only logical to examine how the use of translation technology in the localization of software products impacts the user experience and, hence, the commercial viability of a product.

Large software corporations have implemented MT and post-editing (PE) cycles as part of their localization processes for some time now (e.g. Microsoft, Google and Amazon). As MT technology advances, raw, unedited MT is applied to certain components of the user interface to speed-release to markets with lower translation costs (Schmidtke and Groves 2019). However, it is widely accepted that raw MT contains errors and so, where it is employed, we need to understand how linguistic quality impacts the user experience.

To answer this question, results are presented here from a usability experiment involving Japanese, German, Spanish and English native speakers using the application Microsoft Word while being recorded via an eye-tracker.

## Related work

MT and PE have been implemented in some large organizations since the 1980s (the European Commission and the Pan American Health Organization, for example); however, it is only in the last fifteen years that large corporations have included MT in their standard localization workflows (Plitt and Masselot 2010; Schmidtke 2016). According to the 2019 Language Industry Survey by the EUATC (European Association of Translation Companies),^5^ companies and individual professionals want to increase the use of MT and this technology and associated automated workflows are a clear priority for larger companies.

In reaction to early commercial implementations of MT there was an increase in translation and localization research to find out more about translator interaction with MT in PE (see seminal work from O’Brien 2006; De Almeida and O’Brien 2010; Guerberof 2012; Moorkens et al. 2015). In these studies, the benefits of using MT to increase productivity while not adversely affecting the final quality of the product were established depending on certain constraints such as, logically, the quality of the raw MT output. However, less attention has been paid to the end-user reception of products translated using MT. The reception of  MT output by translators has generally been the focus of empirical studies rather than by end users, and although translators are also a type of user, the commercial user might not necessarily be concerned with the same linguistic aspects as translators.

Some research has tried to fill this gap by analysing the usability of MT in different contexts. Experiments have been designed to ascertain whether users understood instructions translated using MT in comparison to those using either the original or post-edited text (Doherty and O’Brien 2012, 2014; Castilho et al. 2014; O’Brien and Castilho 2016). The results show that usability increases when users read either the original text or text that has been post-edited, even with minimal changes (known as light post-editing), when compared to raw MT output. However, users could complete most tasks using the latter, even if this activity took longer or if the experience was less satisfactory. Results, however, were not equal for all languages tested.^6^

Bowker (2015) studied the difference in user experience when reading text on websites with translatability rules applied (a set of guidelines applied to the source to improve MT). She found that the user experience of source-language readers is less satisfactory when these rules are applied, while that of the target-language readers (Spanish, in this case) improves. As a follow up to this research, Bowker and Buitrago Ciro (2018) replicated this experiment with more participants (Spanish, French Canadian, and Italian) and reported similar findings. When the text was post-edited, however, readers preferred the texts that had been translated without translatability rules applied to the source.

The most extensive research to date on measuring acceptability of machine-translated enterprise content by users was carried out by Castilho as part of her doctoral thesis (2016). In this work, Castilho shows that the level of quality produced through PE has a significant effect on usability for German, Chinese and Japanese users of enterprise content. She also highlights, however, that the raw MT versions were usable, and participants were still able to perform the assigned tasks with these instructions. Because of its relevant content (a Microsoft application) and design, the research described in this paper draws heavily on Castilho’s work.

Castilho and Guerberof Arenas (2018) explored reading comprehension for Spanish and Chinese users when using statistical MT (SMT) and neural MT (NMT) engines to translate an IELTS (International English Language Testing System) test. The authors found that users completed more tasks in less time with a higher level of satisfaction when using translations from the NMT system.

Using a questionnaire, Van Egdom and Pluymaekers (2019) examined how different degrees of MT post-editing (minimal, light, moderate, and full) impacted the user who read two different types of texts (informative and instructive) that had been post-edited. They concluded that different degrees of PE “make a difference” (idem., 168). However, the distinctions between, for example, moderate and full PE was not obvious to the users.

Screen (2019) looked at the English and Welsh language pair. He used an eye-tracker to measure fixations while participants read a post-edited text and a text produced without the aid of MT. After this task, the participants rated the texts according to readability and comprehensibility. He found no statistically significant differences between the two groups.

Other studies have tried to explore how the use of MT can improve or affect communication in collaborative processes in multilingual groups. For example, Gao et al. (2013) explore whether highlighting keywords (i.e. words or concepts that are important) in bilingual communication (English and Chinese) using MT facilitates the communication process. The researchers conclude that highlighting not only brings clarity to the communication but also improves the impressions of the partner and the quality of that collaboration. Further to this, Gao and his colleagues (2014) explore whether consciously using MT in communication has an impact on collaboration between English and Chinese speakers, and they found that, if participants believe that MT was involved, they were less likely to attribute poor communication issues to their partners.

Wang et al. (2013) analyse whether communication between English participants and Chinese participants who have English as their second language could improve by using MT in their native language. The findings suggest that MT helped the Chinese participants to produce ideas in English, but both groups found the English messages that were not mediated by MT to be more comprehensible.

Lim and Fussell (2017) analyse how people understand social posts in languages they do not understand. They found that users not only rely on MT to understand messages, but also on the context of the message by means of visual content (pictures and emojis), contextual and cultural cues, and background knowledge, among others. Further, when reading the MT content, they would focus on keywords to make sense of the overall meaning of the post. They also report that MT could also introduce confusion to the communication when a translation is wrong or obscure.

Pituxcoosuvarn et al. (2018) carry out an ethnographic study in a face to face communication setting to see how children from a multilingual background (Japanese, Khmer, Korean and English) use MT in a workshop to create an animation using clay figures. They find that when the MT message is not understandable, the children employed different strategies, for example: face to face communication in a common language, drawing, gestures or face to face communication using an interpreter as mediator. They also observe that children did not always use MT to communicate; sometimes they resorted to common words between languages or even to the use of objects.

None of the studies above focus on a comparison with an existing human translation. In many cases the language pairs are challenging for MT and often the MT is not described in sufficient detail for the reader to know whether it was customized for its communicative purpose. Studies tend to focus on the impact of using raw MT in collaborative communication and how this might change the perception of the partner and the technology used for this communication rather than on single users working with an application, as in this project. However, they are relevant because raw MT is used in the communication exchange and the studies explore how participants compensate in the communicative situation where MT fails, for example, by using images or context to understand messages.

## Methodology

A within-subject experiment was devised to gather enough target language (TL) data for a statistical analysis to explore the topic of usability and translation modality. To compare the user experience between the translations and the original source, we also devised an in-between subject analysis between the source language (SL) and TL participants. The following sections describe the methodology in more detail.

### Research questions

As mentioned in the introduction, our overall question is *What is the impact of translation modality on the user experience?* To answer this overarching question, the following research questions were devised:

**RQ1:** Will users complete a significantly different number of successful tasks depending on the translation modalities (EN, MT or HT)?
**RQ2:** Will there be significant differences in time in relation to the successful tasks in the different translation modalities (EN, MT or HT)?
**RQ3:** Will the participants have significantly different satisfaction depending on the translation modalities (EN, MT or HT)?
**RQ4:** Will participants expend significantly different amounts of cognitive effort when performing the tasks in different translation modalities (EN, MT or HT)?

### Measuring usability

Following previous studies on MT post-editing usability mentioned in this paper (Doherty and O’Brien 2012, 2014; Castilho et al. 2014; Castilho 2016), usability was defined as per the ISO/TR 16982 guidelines: “the extent to which a product can be used by specified users to achieve specified goals with effectiveness, efficiency, and satisfaction in a specified content of use” (ISO 2002).^7^

*Effectiveness* was measured through task completion. Users were presented with 6 tasks to complete through interaction with different components of the user interface. The more tasks the user completed following specific instructions, the higher the effectiveness percentage was (from 0 to 100%). The difficulty of the task was not given a weight because the intention was to understand the language impact rather than the usability of a feature, so absolute numbers were used. Moreover, the same number of users were exposed to the same number of tasks within the same translation modality to counterbalance the difficulty against the experience of certain users when working on certain tasks. The formula used followed the work by Doherty and O’Brien (2012, 2014):

$$\frac{{{\text{number}}\;{\text{of}}\;{\text{tasks}}\;{\text{completed}}\;{\text{successfully}}}}{{{\text{total}}\;{\text{number}}\;{\text{of}}\;{\text{tasks}}}} \times 100\% = {\text{effectiveness}}$$

*Efficiency* was measured by considering the tasks that were completed in relation to the time it took to complete those tasks. If less time was invested to complete a task, then the efficiency score was higher, and vice versa. We are aware that this formula includes Effectiveness, but we wanted to consider not only the time it took to complete the task, but also the number of successful tasks. Any Efficiency formula that looks to consider these two variables will give a number that might not be meaningful on its own but is if used to compare the translation modalities. The formula used follows that in Doherty and O’Brien (2012):

$$\sum \frac{{{\text{accuracy}}}}{{{\text{total}}\;{\text{task}}\;{\text{time}}\;{\text{in}}\;{\text{seconds}}}} \times 100,$$

$${\text{where}}\;\frac{{{\text{task}}\;{\text{successes}}}}{{{\text{total}}\;{\text{tasks}}}} \times 100 = {\text{accuracy}}$$

*Efficiency* was also measured in terms of cognitive effort using an eye-tracking device. Following Castilho (2016) we looked at fixation duration (total length of fixation in an area of interest or AOI), fixation count (total number of fixations within an AOI), visit duration (the duration of each individual visit within an AOI in seconds) and visit count (number of visits to an AOI). This is counted between the first fixation on the active AOI and the end of the last fixation within the same active AOI where there have been no fixations outside the AOI. Eye-tracking has been established as an adequate tool to measure cognitive effort in MT studies (as initially confirmed by Doherty and O’Brien 2009 and Doherty et al. 2010). See Sect. 3.7 for specifications of the eye-tracker used in the experiment. The type of equipment posed certain constraints on other measurements (for example, pupil dilation or saccades) that were not used in this project.

*Satisfaction* was measured through the IBM After-Scenario Questionnaire (Lewis 1995) containing a series of statements that users rated. This questionnaire was chosen instead of other frequently used questionnaires such as the Software Usability Scale (SUS; Brooke 1996) or Post-Study System Usability Questionnaire (PSSUQ; Lewis 1992) because, in this project, only a subset of tasks was assessed while in the other questionnaires an entire system is rated. The ASQ has three questions to rate on a 7-point Likert-type scale, where 1 represents ‘strongly agree’ and 7 ‘strongly disagree’. This questionnaire was modified to address the language factor in two questions to differentiate between the quality in the instructions and that in the application, MS Word. The result was as follows:

1. Overall, I am satisfied with the ease of completing the tasks in this scenario.
2. Overall, I am satisfied with the time it took to complete the tasks in this scenario.
3. Overall, I am satisfied with the instructions given for completing the tasks.
4. Overall, I am satisfied with the language used in the Word menus, dialog boxes and buttons.

Question 3 was added, even if it does not refer to MS Word specifically, because participants always worked with the Instruction window visible while working with MS Word (see Fig. 2). It was, therefore, important to differentiate the language used in both windows.

Since each translation modality (HT, MT, EN) represents a scenario within the software application (as per the questionnaire), both terms are used interchangeably in this paper.

#### Retrospective think aloud

Once the participants had completed the tasks, the gaze data was replayed, and they were asked to comment on what they were doing, thinking or feeling during the experiment. The participants were recorded using Flashback Express 5. The retrospective interviews took approximately 15 min. One researcher asked certain questions to elicit responses from the participants, such as ‘How did you find this task?’, ‘What were you thinking at this point?’, ‘How was the language in this menu?’, ‘Had you done this task before?’, or ‘Did you notice any difference in Word when you came back from the break in the experiment?’

### Content and design

In collaboration with Microsoft Ireland, MS Word was chosen as the optimal application for the experiment. This was firstly because the study sought to reach as many participants as possible and MS Word is the most popular application in the suite, and secondly, because it was important to measure the impact of the translation modality as opposed to the users’ computing knowledge and skills, and MS Word is a relatively easy application to use. The software version used was Microsoft Word 2016 MSO (16.0.9126.2315) 32-bit in English and it was changed to the different languages using Word/Options/Language/Change display language.

These language versions are a result of the translation delivered by language service providers to Microsoft. As mentioned above, it is relevant to note that the localization process might involve translating a segment of text without any technological intervention, but, in general, it includes the aid of MT and translation memories,^8^ among other reference material, as well as a review cycle. At the time of this experiment, all software strings were processed by translators in Microsoft’s localization process.

A specific version of MS Word was created for the MT scenarios, translated from English using the business partner’s highly customized Microsoft Translator SMT engine (Quirk et al. 2005).^9^ At the time of implementing this experimental setup, customized Microsoft NMT engines were not available. This SMT engine is highly customized so the quality was high enough to be implemented as part of the localization workflow in the organization (Schmidtke 2016).

A warm-up task and 6 subsequent tasks were selected. The criteria for task selection was that the tasks contained enough text, i.e. that they always had at least one word to select in a series of steps, to measure the translation modality, that they were coded for telemetry purposes (for a second phase of this experimental project dealing with telemetry and translation), that they were present in all the languages tested (German (DE), Spanish (ES), Japanese (JA) and English (EN)), and that they were relatively new or non-standard to minimize the effect of previous user experience or familiarity with the tasks. For this reason, the users were not allowed to use the Help or Search functions in Word during the experiment; the intention was that the users would navigate the application and use the UI text to reach the task goals (see Appendix 1). It is important to highlight that, although we devised one warm-up task and 6 tasks, the whole MS Word application was presented in two modalities: Human Translated and Machine Translated depending on the group (see Sect. 3.4). The warm-up task involved selecting a paragraph and changing the font. The other six tasks were:

1. Selecting a digital pen and drawing a circle using a defined thickness and colour
2. Changing the indentation and spacing for the paragraph (presented to the users)
3. Automatically reviewing the document (Spell checking)
4. Selecting the option Frequently confused words from the Word Options/Proofreading box in the File menu
5. Inserting a section break
6. Finding the Learning Tools option in the corresponding menu and changing the page appearance.

The task instructions were evaluated by an English native speaker, writer and researcher from the Connect Centre at Trinity College Dublin to assess comprehension of the instructions and the environment. These were then translated by Microsoft language service providers into German, Japanese and Spanish. They translated the texts following specific instructions to respect the fluency and accuracy of the text and the experimental design (e.g. to translate repeated terms consistently and not replace them with pronouns, not to use the names of menus, dialog boxes or options if they were not present in the source text, to integrate the options within the text and not to use upper case, etc.)

#### Machine translation quality

It was not possible to analyse the original and translated texts (both human and machine translated) with standard readability metrics, nor was it possible to do a straight comparison of the software files because of the way the MS Word application is built, thus making it difficult to isolate the text, extract the strings and compare the software files in different translation modalities (SL and TLs). Therefore, Japanese, German and Spanish native speakers who were language lecturers and language researchers at Dublin City University (DCU) evaluated the tasks in the released versions and compared them manually to the raw MT environment. These evaluators commented on the high quality of the MT versions, and they highlighted the sentences and words that were not idiomatic, those that were wrong, and those that were different from the released version in the 6 tasks selected. Table 1 shows the issues found in the MT versions for the 6 tasks in the project. The English in brackets is provided as reference; the bold options indicate errors in the MT output.

**Table 1:** Sample of errors found per task in the MT version

Tasks	EN	JA	DE	ES
1 HT	Draw	描画 (Draw)	*Zeichnen* (Draw)	*Dibujar* (Draw)
1 MT		図形の調整 (Arranging shapes)	*Zeichnen* (Draw)	*Dibujar* (Draw)
2 HT	Right (as in Right Indent)	右 (Right)	*Rechts* (Right)	*Derecha* (Right)
2 MT		そうです (Correct)	***Richting*** (No meaning)	***Correcto*** (Correct)
3 HT	Review	校閲 (Text Correction) スペルチェックと文章校正 (Spellcheck & Text Correction)	*Überprüfen* (to check) *Rechtschreibung und Grammatik* (Spelling and Grammar)	*Revisar* (Review) *Ortografía y gramática* (Spelling and Grammar)
3 MT		レビュー (Review) スペルチェック&文法 (Spellcheck & Grammar)	*Überprüfung* (noun instead of verb) *Rechtschreibung & Grammatik* (Spelling and Grammar)	*Revisión* (noun instead of verb) ***Ortografía & gramática*** (Spelling & Grammar, ampersand is not correct in Spanish)
4 HT	Proofing/Frequently confused words	文章校正 (Text correction) よく間違う単語 (Frequently confused words)	*Dokumentprüfung* (Proofing) *Häufig verwechselte Wörter* (Frequently confused words)	*Revisión* (Revision) *Palabras que se confunden frecuentemente* (Frequently confused words)
4 MT		校正 (Correction) 頻繁にされやすい単語 (Words that are easy to frequent)	*Rechtschreibprüfung* (Spellcheck) ***Häufig verwechselt Wörter*** (Often words are confused)	*Corrección* (Correction) ***Palabras con frecuencia confuso*** (Words often confusing, wrong gender agreement)
5 HT	Section Break	区切り (Section break)	*Umbrüche* (Breaks)	*Saltos* (Breaks)
5 MT		改 (New)	***Zeilenumbrüche*** (line break instead of section break)	*Saltos* (Breaks)
6 HT	Learning Tools Immersive	学習ツール (Learning Tools) イマーシブ (Immersive)	*Lerntools* (Learning tools) *Plastisch* (Vivid)	*Herramientas de aprendizaje* (Learning tools) *Inmersivo* (Immersive)
6 MT		学習ツール (Learning tools) 没入感 (Immersion)	***Lernhilfen*** (Learn Help)/***Beeindrucke*n** (means Impress instead of Immersive)	*Herramientas de aprendizaje*/***Envolventes*** (Immersive, figurative)

As described later, during the RTA (see Sect. 5), some participants did notice these errors (Table 1) because they were required to perform a task that involved reading and selecting these options, while others did not. Depending on their experience and problem-solving strategies, they overcame (or did not) the difficulties posed by these language errors as we will analyse in the discussion of results (see Sect. 4).

### Scenarios and groups

The JA, DE, and ES participants were assigned to two groups. In Group 1, they completed three tasks as (A) HT, and three tasks as (B) MT, while Group 2 was presented with the same tasks but in reverse order, that is, the first three tasks as (B) MT, and the last three tasks as (A) HT. This served to counterbalance the within-subject effect. Between scenarios there was a brief pause that allowed the researcher to change the MS Word configuration and recalibrate the eye-tracker. Because this pause to change the application was needed and the 6 tasks were presented in both modalities in equal numbers, they could not be automatically counterbalanced in the eye-tracker as we needed to manually control for the translation modality. The participants knew that they were taking part in an MS Word usability experiment but were blind to the fact that MT was being used in three tasks. The EN group was presented with a warm-up task and 6 other tasks as well. As with the other groups, they had a brief pause between the tasks.

### Pre-task questionnaire

The participants were asked to fill in a questionnaire before the experiment. The questionnaire assessed the experience users had in using the word-processing application MS Word, their native language and their level of English, gender, age, education level, as well as their experience in doing the tasks that were part of this experiment. The questionnaire was completed online using Google Forms.

### Participants

The criteria for inclusion of volunteer participants was that they were native speakers, that they were willing to participate in the research and sign a consent form, and that they were frequent users of word-processing applications. The participants were recruited through advertisements in social media and email lists within DCU, although participation was not limited to students or people associated with the university. The participants were offered a €20 voucher for their contribution. All participants received a Plain Language Statement and signed an Informed Consent form before the experiment (DCU Research Ethics reference REC/2017/200). The experiment took place between August and December 2018.

84 participants took part in the experiment but data from only 79 participants was analysed and is presented here. Some data were discarded due to changes in the original set-up (MS Word version was updated accidentally). Other participants were discarded only from the eye-tracking data due to poor recording quality (see Sect. 3.7). The total number of participants per language is: 18 EN, 22 JA, 19 DE and 20 ES.

#### Description of participants

72% of participants identified as women and 28% as men. Although, more women participated than men, the gender distribution across languages shows no significant differences. The age distribution was 73% in the 18–24, 14% in the 25–34 and 13% in the 35–44-year-old bracket. The age of most participants ranged from 18 to 24, the distribution across languages shows no significant difference. If we consider gender and age, the data is comparable across all languages. The participants were asked to take a test to measure their English level using Lextale (Lemhöfer and Broersma 2012). The mean values were EN 94.58; DE 85.92; ES 67.36 and JA 65.17 out of 100 (the equivalent with the Common European Framework of Reference for Languages the EN and DE group would be classified with the C2/C1 level, while the ES and JA group with B1/B2). There is a statistically significant difference between language groups in their English level (H(3) = 45.27; *p* = 0.00). Post hoc comparisons using the Mann–Whitney Test show significant differences between the JA and the DE (U = 42; *p* = 0.00) and to EN groups (U = 9.50; *p* = 0.00), but not with the ES group.

When participants were asked about their experience using MS Word, there were statistically significant differences between the language groups regarding the length of time the participants had been using word-processing applications (X^2^(6) = 36.23, *p* < 0.001); but this was only true if the JA group was included; otherwise, there were no statistically significant differences between the other languages. When participants were asked to rate their level of proficiency (i.e. “How would you describe your level of proficiency when working with word-processing applications?”) using a 5-point Likert scale (1 being Novice and 5 being Very proficient), the average values were: JA = 2.14; DE = 3.37, ES = 3.25 and EN = 3.83. A statistically significant difference (H(3) = 35.67, *p* < 0.001) exists between the users per language group when reporting their experience.

**Fig. 1:**
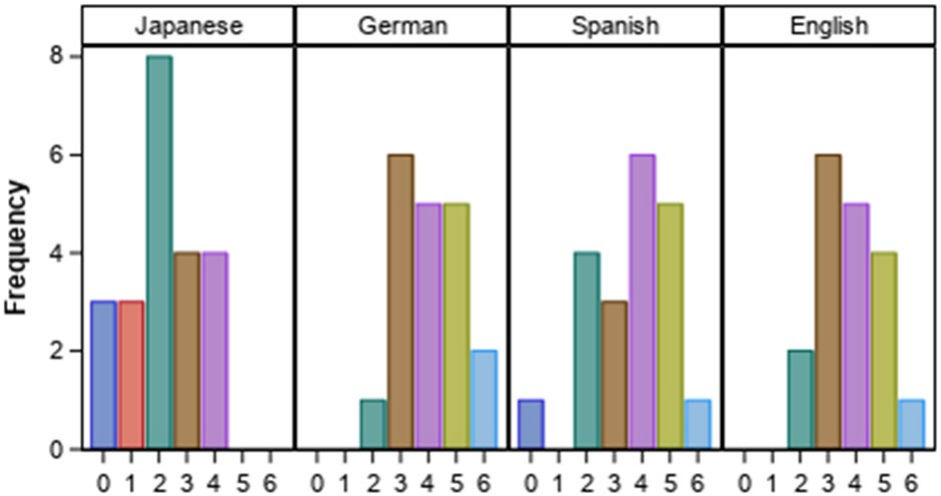
Experience in the 6 experimental tasks per language

Finally, participants were asked about their experience in the 6 experimental tasks that they were going to be presented with during the experiment. They were simply asked to mark the tasks they were familiar with within 10 word-processing tasks; these 10 tasks included the 6 experimental tasks. Figure 1 shows the results per language.

The Japanese group (JA) reported that they had an average experience of 2.14 tasks out of the 6 tasks; the DE group reported an average of 4.05 tasks out of 6; the ES an average of 3.60 tasks; and finally, the EN group reported an average of 3.78 tasks out of 6. There are statistically significant differences (H(3) = 20.84, *p* < 0.001) in the distribution of the variable Percentages_of_tasks_in_experiment according to the language group. If the JA group is not considered, however, there are no statistically significant differences among the language groups. As can be observed in this description, and as was observed in our preliminary results that focused on the Japanese users (Guerberof et al. 2019), the JA group shows significantly lower experience than the other language groups.

### Experimental setup

The data recording equipment consisted of a Tobii T60 XL wide screen eye-tracker with a 24-inch monitor and 60 Hz sampling rate and a laptop computer (Intel Core 1.7 vPro^tm^, 2.00 GHz 2 Core, 4 Logical processors, 8 GB RAM). The laptop was used for stimulus presentation and eye-movement recording. The stimuli were presented with a 1600 × 900 resolution. The software used to record and analyse the data was Tobii Studio 3.4.5 1309, Professional Edition. The fixation filter selected was an IV-T Filter provided by the manufacturer. The filter has a velocity threshold of 30 degrees, a maximum time between fixations of 75 ms and a maximum angle of 0.5°. Fixations under 60 ms were discarded.

The participants were calibrated using a nine-point calibration screen (automatic). The participants were recalibrated if the Tobii system reported a poor calibration or if the calibration points were not clearly defined within the grid. The optimal distance to the eye-tracker was set at 67 cm. However, this varied as the participants were not required to use a chin rest to preserve ecological validity during the experiment.

**Fig. 2:**
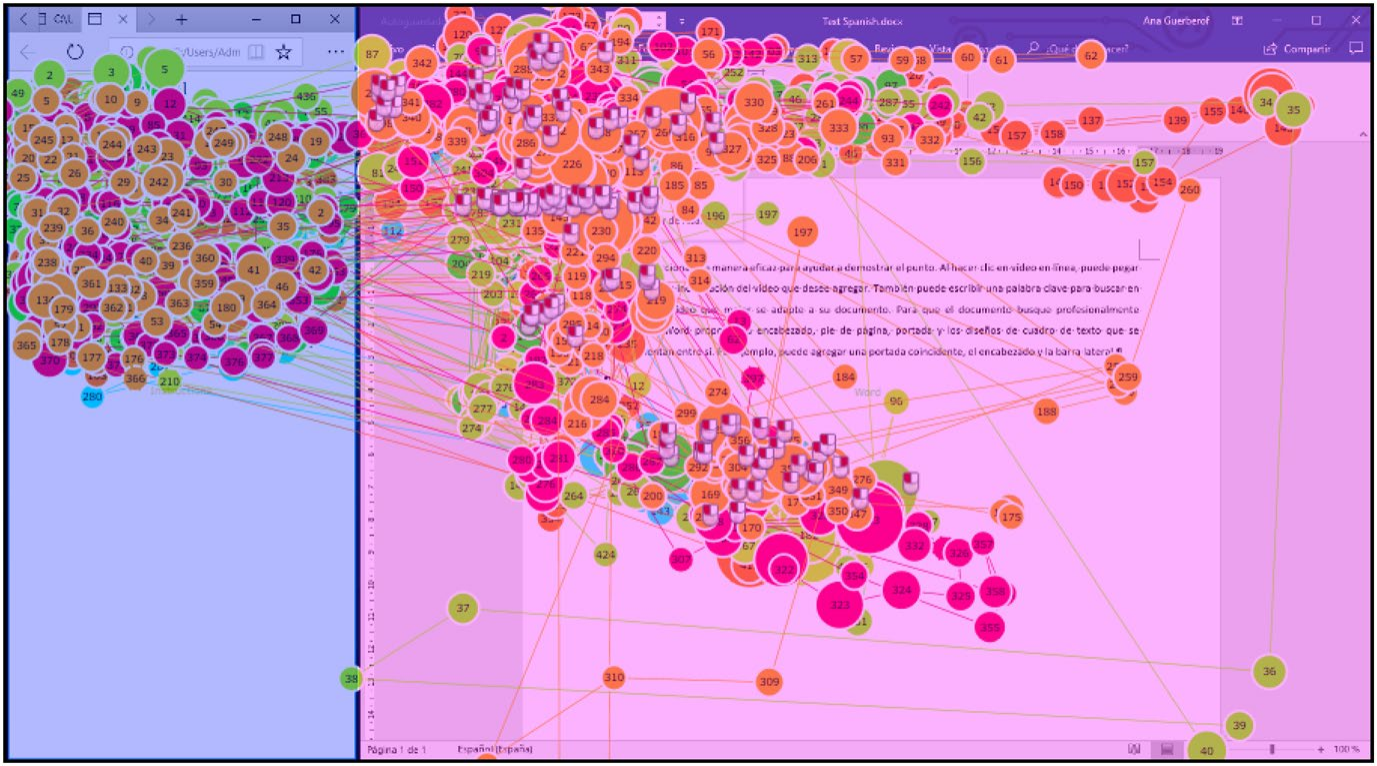
AOIs and fixations window

To estimate the cognitive effort using an eye-tracker, two Areas of Interest (AOIs) were defined. One AOI was the Instructions window (26%, 369,516 px) and the other AOI covered the MS Word application window (74%, 1,065,165 px) to determine whether participants would consult the Instructions window more often if they did not understand the text in the MS Word window when using different translation modalities. One participant in the ES group moved the screens slightly, therefore 6 ES and 2 JA participants had slightly different AOIs sizes for the Instructions (23%, 328,500 px) and for the MS Word application (77%, 1,107,000 px). Figure 2 shows the experimental setup with the two AOIs highlighted in blue (Instructions) and pink (Word application).

To test the quality of the sample, the gaze data in the Tobii system and the velocity charts were checked. Moreover, the segments that represented one task per participant were exported to calculate the eye validity codes within these six segments. A minimum of 80% gaze sample was required for a recording to be considered valid. This meant that each participant had at least one eye or both eyes on the segments 80 per cent of the time.

### Statistical methods

To analyse the results statistically, SAS v9.4 and IBM SPSS Statistics, v24 were used. The decisions were made with a significance value of 0.05.

For each of the variables explored, we include a bar chart showing the mean per language and scenario and the confidence interval for this mean.

To determine the effect of the scenario (HT, MT and EN) for the response variable Effectiveness, Efficiency and Satisfaction, a linear mixed model was calculated according to the scenario and task groups (1, 2, 3 vs. 4, 5, 6) and the interaction between scenario and language (Type III Test). The tasks and scenarios are considered fixed factors and the repeated measures of each participant are included in the model (random effects). For Time, a linear mixed model of the logarithm transformed variable ‘Total time in seconds’ was calculated.

The adjustment for multiplicity was Tukey by default for all variables when the languages were compared with each other. When the categories were compared with the English group (EN), the Dunnett–Hsu adjustment for multiplicity was used.

All variables (Effectiveness, Efficiency, Time, and Satisfaction) were contrasted to see if there were statistically significant differences in the order the scenarios were shown to the TL participants. As expected because of the within-subject design, there were no statistically significant differences according to the order in which each scenario (HT and MT) was carried out.

## Results

### Effectiveness

**Fig. 3:**
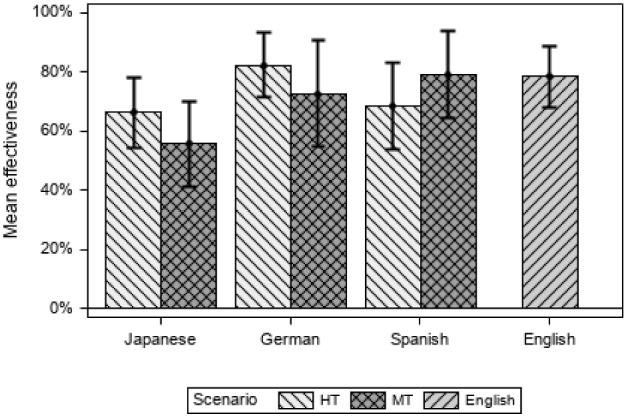
Mean Effectiveness according to scenario and language

The variable Effectiveness represents the percentage of tasks completed. Figure 3 shows this variable according to the language and scenario based on the mean values and the confidence interval for this mean.

Figure 3 shows that the DE group has the highest task completion percentage overall in the HT scenario, followed by the ES MT scenario (both higher than the EN group). The JA group has the lowest Effectiveness percentages in both scenarios compared to all the other language groups (see Appendix 2 for a table with descriptive values). As we saw in the description of participants (Sect. 3.6.1), the JA group has the least experience, so it seems logical that when confronted with new tasks, the participants in this group completed fewer tasks than the other more experienced groups. Surprisingly, the ES group shows higher effectiveness when working in the MT scenario. This could be explained by the quality of MT in the English to Spanish language pair. The pair English-Japanese shows lower performance in statistical MT and it is known to be a more challenging language combination than the other languages involved in this experiment (Doddington 2002).

These results do not appear to be clearly favourable to HT or even favourable to the original EN version. A linear mixed model was calculated for the group of tasks (1, 2, 3 vs 4, 5, 6), the scenario (HT, MT) and the interaction of language and scenario (JA, DE and ES) including repeated measures at participant level (random effect). This shows that there are statistically significant differences between tasks (F(1, 57) = 69.37; *p* < 0.001), and considering the interaction of language and scenario (F(4, 57) = 3.33; *p* = 0.016), but not solely between scenarios. Although the differences between the two sets of tasks was not intentional during the experimental design, it became apparent when running the experiment in the laboratory that the second set of tasks were more difficult for all languages and this was especially true for Task 4 (Word/Options).

For the interaction between language and scenario, there are statistically significant differences between the modality DE HT and JA MT (*t* = 3.25, *p* = 0.002). There are no significant differences between the other language groups and scenarios. Notwithstanding, DE HT is more effective than DE MT (11.36%), JA HT is more effective than MT (7.64%), but ES MT is more effective than ES HT (10.42%).

When the English group (EN) is compared to the other languages, statistically significant differences are observed between tasks (F(1, 75) = 90.94; *p* < 0.001) and considering the interaction of language and scenario (F(4, 75) = 3.36; *p* = 0.014), but not between scenarios. In this case, there are statistically differences between the pair JA MT and EN (*t* = 2.91, *p* = 0.024). The JA MT group is less effective than the EN group.

It appears that the experience of the JA participants might have had an impact on their effectiveness measurement. To see how these two variables were related, we ran a Pearson coefficient for the variables P_tasks_in_the_experiment (the percentage of tasks the participants reported they could do prior to the experiment) and Effectiveness. The results show a significant positive correlation (r(77) = 0.42 *p* < 0.001) considered weak to moderate indicating that the higher the percentage in experience, the higher the Effectiveness in the language groups.

While experience does moderately explain the variable Effectiveness, we know that the type of task is also an important factor (participants were significantly less effective for tasks 4, 5 and 6) and it also seems that the translation modality was especially significant for the JA group when compared with other language groups: the MT scenario had *a slowdown effect* in the JA group if compared with the DE and ES groups (see Guerberof et al. 2019) for a detailed description of the findings from the Japanese group).

In a similar study, Doherty and O’Brien (2014) found that English, Spanish and German participants had higher task completion scores than their Japanese group, and these were significantly higher for the English and Spanish groups. Castilho (2016), in her study involving Japanese, German, and Chinese participants working with tasks in Excel, also found that the Japanese group had lower Effectiveness scores than the German group. She found that the German and Japanese groups were more effective when working with the human-translated instructions as opposed to the MT instructions in her experiment. However direct comparisons with these previous experiments are not possible since in the previous experiments MT was used to translate the instructions used to complete the tasks and not for completion of the tasks themselves as in this experiment.

### Efficiency

**Fig. 4:**
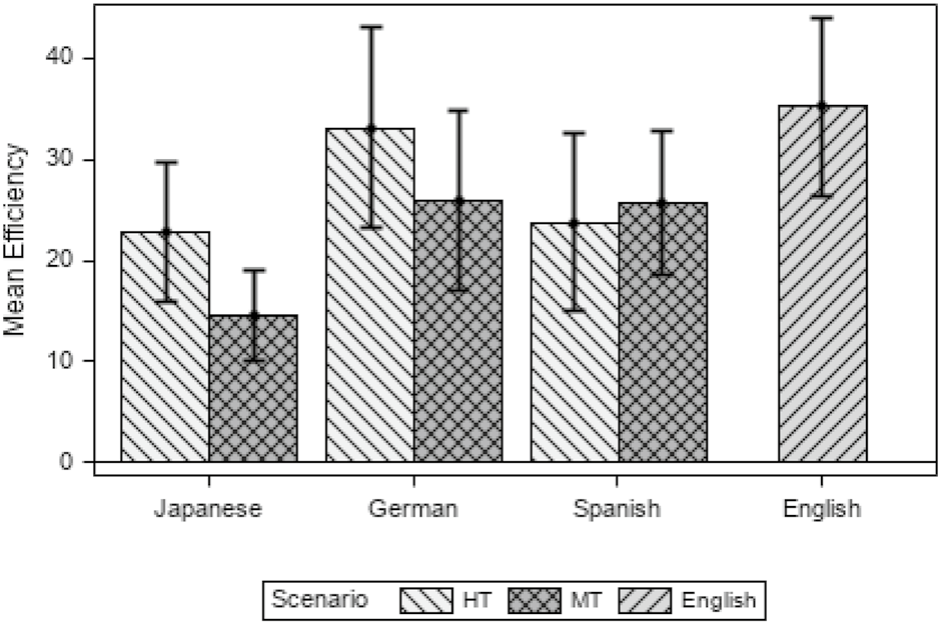
Efficiency by language and scenario

Efficiency also considers the tasks completed but it factors in the time spent on completing them as seen in the formula (Sect. 3.2). The higher the Efficiency score, the more efficient the language group is as shown in Fig. 4.

The bar diagram shows that the variable Efficiency is highest in the EN group. The DE and JA groups have higher Efficiency scores in the HT than in the MT scenario. Here again, the ES group has slightly higher Efficiency scores in MT than in HT. The JA group has the lowest Efficiency scores in both HT and MT compared to all the other languages in all scenarios (see Appendix 2 for a table with descriptive values).

A linear mixed model was calculated for the variable sqrt(Efficiency) according to the group of tasks (1, 2, 3 vs 4, 5, 6), the scenario (HT vs. MT) and the interaction of language and scenario (JA, DE and ES) including repeated measures at participant level (random effect). The response variable Efficiency was transformed by the square root because some values were very low (for example for those participants that had not completed any tasks, the value was 0) and the distribution was asymmetrical. Once the variable was transformed, the data was normal with a slight negative skewness. The results from the model show that there are statistically significant differences between scenarios (F(1, 57) = 4.57; *p* = 0.037), tasks (F(1, 57) = 131.83; *p* < 0.001) and in the interaction of language and scenario (F(4, 57) = 3.76; *p* = 0.009).

This means that if we consider only the translation modality (HT vs. MT), participants are significantly less efficient when using MT (*t* = 2.14, *p* = 0.037). Participants were significantly more efficient in tasks 1, 2 and 3 than in tasks 4, 5, 6 (*t* = 11.48, *p* < 0.001). If the interaction between language and scenario is considered, statistically significant differences are found between the pair DE HT and JA MT (*t* = 41; *p* = 0.002).

If the English group (EN) is included in the model, there are statistically significant differences between scenarios (F(2, 75) = 5.27; *p* = 0.007), tasks (F(1, 75) = 176.54; *p* < . 0001) and the interaction of language and scenario (F(4, 75) = 3.27; p < 0.0158). In this case, the EN group is significantly more efficient than MT (*t* = 2.97, *p* = 0.011) and if the language and scenario is considered EN is significantly more efficient than JA MT (*t* = 3.81, *p* = 0.002).

Therefore, the participants working with the EN version are more efficient overall. This might suggest that “translation”, regardless of modality, has a negative impact on usability, at least when efficiency is calculated. Also, the participants are more efficient in the HT than in the MT scenario overall. And if all the language and scenario categories are examined, the participants in the JA MT scenario are the least efficient. The results from the Pearson correlation are logically very similar to those found in Effectiveness (r(77) = 0.4, *p* < 0.001) indicating that the higher the percentage in experience, the higher the Efficiency in the language groups.

**Fig. 5:**
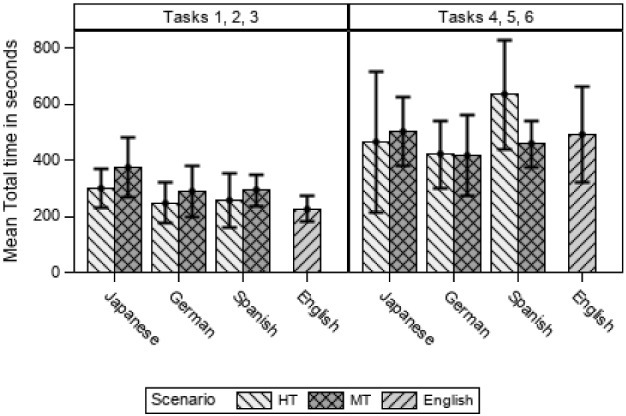
Mean total time according to scenario, language and tasks

Figure 5 shows the results for the variable Time on its own without considering the variable Effectiveness according to language, scenario and tasks.

Figure 5 shows clearly that tasks 1, 2 and 3 took less time than the second set of tasks. In the first three tasks, participants spent more time in MT for all languages. However, for the most difficult tasks (4, 5 and 6), the results are not equal for all languages. A linear mixed model of the logarithm Total time in seconds according to tasks, scenario, and the interaction between language and scenario finds that there are significant differences between tasks (F(1, 75) = 90.87, *p* < 0.001), but not between scenarios or the interaction between scenarios and languages when time is analysed.

In a similar study, Doherty and O’Brien (2014) found that the English group was also significantly more efficient than the Japanese and the German groups but not when compared with the Spanish group. Castilho (2016) in her doctoral study also found that the English group was statistically more efficient than the other groups while the other languages were not significantly different.

### Cognitive effort

**Fig. 6:**
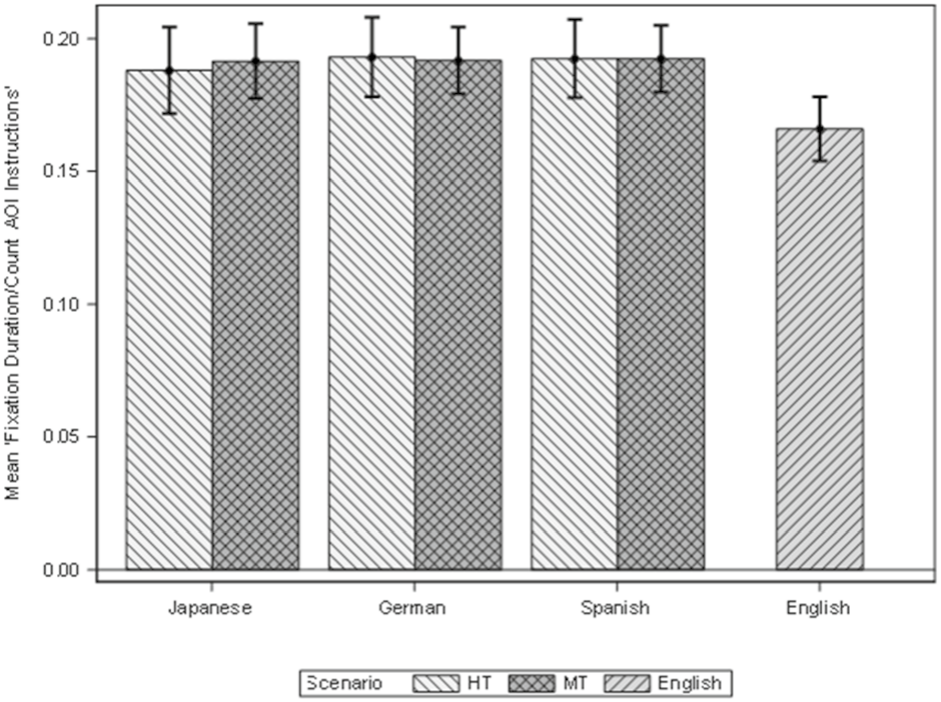
Fixation duration instructions AOI

**Fig. 7:**
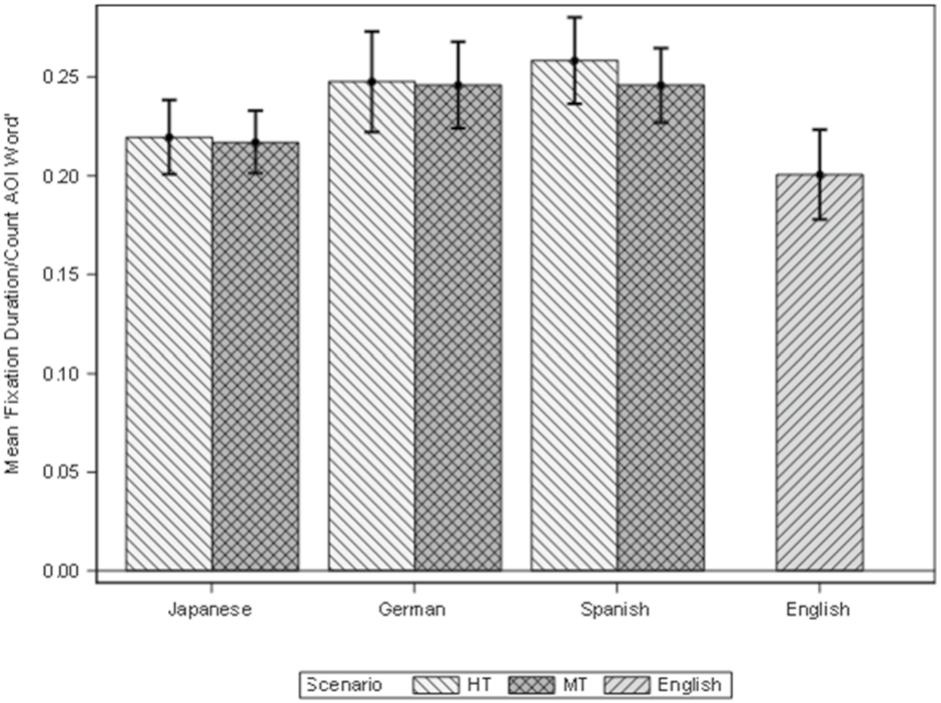
Fixation duration MS Word AOI

As explained in the methodology section, the variables Fixation duration, Fixation count, Visit duration and Visit count were calculated in two different AOIs, Instructions and MS Word (see Fig. 1). Descriptive data for all these variables is included in Appendix 3. We are presenting here the results for the variable Fixation Mean for these two AOIs. The Fixation mean represents the Fixation duration in seconds divided by the number of fixations, Fixation count. Figures 6 and 7 show the Fixation mean for the Instructions (left) and for MS Word (right) AOIs according to the three different scenarios, and the four languages.

Figures 6 and 7 show that the EN group has a shorter fixation mean in both the Instructions and the MS Word AOIs, and this could point to the fact that since both the instructions and the application were originally written in English, this made reading and identifying options less cognitively demanding for the EN group when compared with other (translated) languages. We have also seen that the EN group was the most efficient group; it took the participants in this group less time to complete more tasks. If we look at the Instructions AOIs, the EN group have the lowest mean (0.17). The rest of the languages in all the modalities have the same fixation mean (0.19). This means that their cognitive effort when reading the instructions was similar for all participants.

In the MS Word AOIs, the EN group shows again the lowest fixation mean (0.20), followed by the JP group in both modalities (0.22), and the DE group in both modalities (0.25). The ES group shows the highest mean in the HT scenario (0.26) while the MT scenario shows a mean comparable to the DE group (0.25). The surprising result is that the JP group appears to have a lower cognitive effort, if we take the mean fixation into consideration, than the ES and DE group even if they appeared to have struggled more with the tasks. We believe that this has two explanations. On the one hand, the JP group completed fewer tasks, so they spend less time in the Word AOI, when they could not complete a task, they simply move on to the next task. On the other hand, the fixation mean represents the division between fixation duration and count, hence here the divisor, the count, is higher than the duration, the JP group shows a higher number of fixations than the duration of these fixations, and this indicate that JP group concentrated less on a task and more in looking for that task.

This can also be seen in the pattern of lower Fixation mean values for the second set of tasks (4, 5, 6) than in the first three (1, 2, 3) even if, as we have seen throughout the study, the second set of tasks was more difficult to resolve for all participants. Hence, we believe that the fixation duration might be a more representative measure of cognitive effort in this case. See Appendix 3 for complementary measurements on duration.

Having clarified this aspect, we can also see that the modalities, except for the EN group, do not show visible differences, that is, differences between HT and MT in cognitive effort are not visible by looking at the fixation mean.

To explore the significance of the values, a linear mixed model for the logarithm Fixation Mean for the AOI Instructions, according to the group of tasks, scenarios and interaction between language and scenario (for the JA, DE and ES groups) found that there were statistically significant differences only when the tasks are considered (F(1, 53) = 4.82; *p* = 0.032). But also, the logarithm Fixation Mean for the AOI MS Word shows significant differences according to tasks (F(1, 53) = 90.6; *p* < 0.001). However, the scenarios show no significant differences.

Secondly, we explore the effect of having the EN group in the model for logarithm Fixation Mean for AOI Instructions, and there are statistically significant differences between the scenarios (F(2, 71) = 6.42; *p* = 0.003). The EN scenario shows significantly lower Fixation Mean than the HT and MT scenarios. This means that the English group fixated for significantly time in the instructions than the rest of the language groups (as in Doherty and O’Brien 2014). But also, whilst the logarithm Fixation Mean for the AOI MS Word shows significant differences according to scenarios (F(2, 71) = 7.66, p = 0.001 and tasks (F(1, 71) = 137.85; *p* < 0.001), the EN group shows a lower fixation mean in comparison to the other language groups.

### Satisfaction

**Fig. 8:**
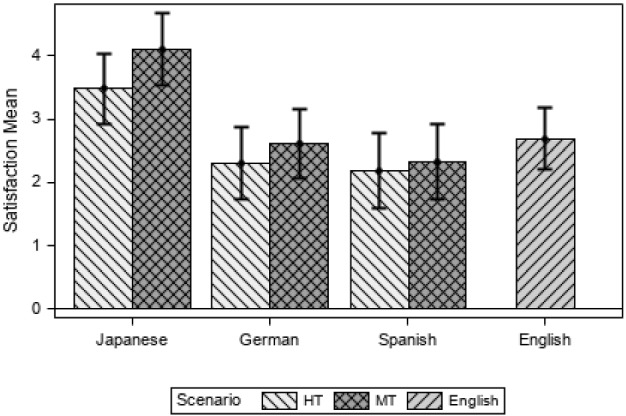
Satisfaction mean according scenario and language

Satisfaction was calculated using the four questions from the two post-scenario questionnaires that were ranked per participant on a 7-point Likert-type scale where 1 indicated the most satisfaction and 7, the least (as defined in the ASQ questionnaire by Lewis 1995). Figure 8 shows the mean for the variable Satisfaction according to scenario and language.

All participants report less satisfaction in MT than in HT. The JA group is the least satisfied and the ES the most satisfied if compared to the DE and EN groups (see Appendix 2 for a table with descriptive values). This result is surprising when it comes to the ES group, since participants were more efficient and effective when using MT than HT. DE and ES are more satisfied than the EN group, and this is also surprising, as we would have expected that participants working with the source language, and showing more efficiency and a lower cognitive load, to be more satisfied (as in Doherty and O’Brien 2014 ) where English had the highest ratings in comprehension, satisfaction and recommendation), but this could point to the fact that different cultures and individuals rate satisfaction differently and that the ES participants, regardless of their performance, noticed linguistic problems in the MT scenario (see Sect. 4.5).

A linear mixed model shows that there are statistically significant differences for the variable Satisfaction between scenarios (F(1, 57) = 5.90; *p* = 0.018), tasks (F(1,57) = 40.81; *p* < 0.001), and the interaction between language and scenario (F(4,57) = 7.72, *p* < 0.001). The estimated mean of Satisfaction is 2.62 in EN, 3.41 in HT and 3.96 in MT scenarios (a lower score indicates a higher satisfaction).

**Fig. 9:**
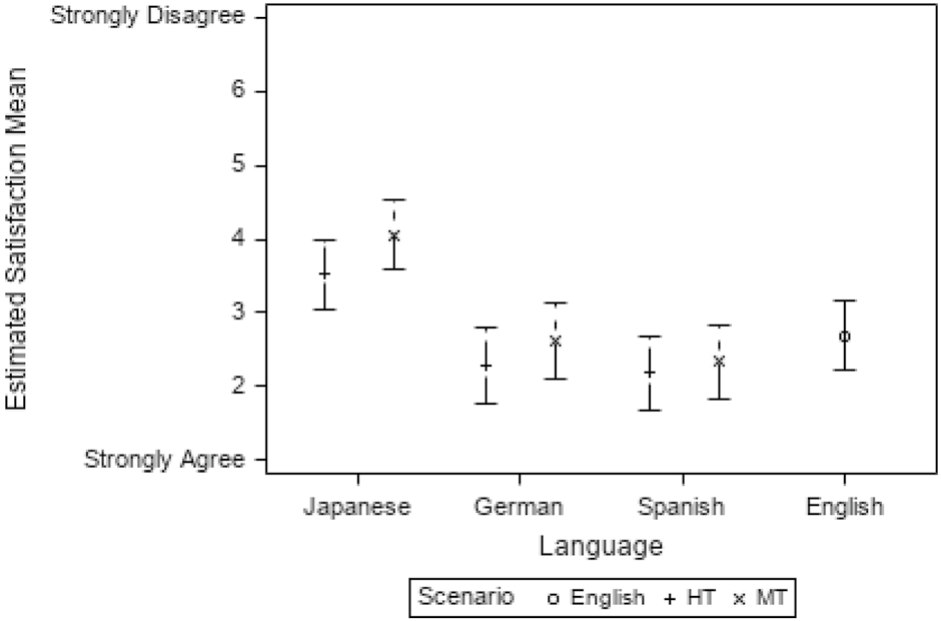
Estimated satisfaction mean per language and scenario

If we consider only the translation modality (HT vs. MT), participants are significantly less satisfied when using MT (*t* = 2.43, *p* = 0.018), the difference in the estimated Satisfaction mean being 0.35, CI_95%_ = [− 0.63, − 0.06]. Logically, as with all the other variables, participants are significantly more satisfied in tasks 1, 2 and 3 than in tasks 4, 5, 6 (*t* =  − 6.39, *p* < 0.001). To explore the behaviour according to language group, the interaction between language and scenario is explored and statistically significant differences are found between the pair DE HT and JA HT (*t* = -3.47; *p* = 0.012). Also, the contrasts between JA HT and JA MT and the other languages and language modalities show statistically significant differences, as the JA group is always significantly less satisfied (see Fig. 9).

When the English group (EN) is included in the model with all the language groups, similar results are obtained, i.e. statistically significant differences between scenarios (F(2, 75) = 3.32; *p* = 0.042), tasks (F(1, 75) = 62.30; *p* < 0.001) and considering the interaction of language and scenario (F(4, 75) = 7.80; *p* < 0.001). In this case, HT has lower values (indicating more Satisfaction) than MT (*t* =  − 2.52, *p* = 0.037) and if the language and scenario are considered, and EN is compared to all the other categories, the JA MT category is significantly less satisfied than the EN group (*t* = 4.09, *p* = 0.001), as Fig. 9 also illustrates.

We wondered if it could be possible that participants who had completed fewer tasks would be less satisfied; a Spearman correlation shows a significant negative correlation (r_s_(77) =  − 0.29; *p* = 0.009), meaning that participants who completed more tasks were also more satisfied, although this is a weak-to-moderate correlation.

We also saw in Sect. 4.1 (Effectiveness) that the experience in doing these 6 tasks had a significant positive correlation, although moderate; logically, the higher the level of experience, the more tasks participants completed. Therefore, we can infer that participants who had more experience and completed more tasks were more satisfied overall, even though this is not the only factor to consider. The scenarios and the tasks were also a factor to consider, and this is particularly true with the JA group.

Finally, if we look at the question that specifically addressed the language in the post-scenario questionnaires (“Overall, I am satisfied with the language used in the MS Word menus, dialog boxes and buttons.”) for the JA, DE and ES groups regardless of the tasks performed, participants are more satisfied in the HT (M = 2.64) than in the MT scenarios (M = 3.28). A Wilcoxon signed rank test shows that HT ranks significantly lower than the MT scenario (Z =  − 3.19, *p* = 0.001) indicating a higher Satisfaction. The results show that 27 participants are more satisfied with HT; 10 participants with MT, and in 24 cases MT was ranked alongside HT. However, when the other questions are compared between scenarios (“Overall, I am satisfied with the ease of completing the tasks in this scenario”, “Overall, I am satisfied with the time it took to complete the tasks in this scenario” and “Overall, I am satisfied with the instructions given for completing the tasks”) no significant differences are found between the language groups, although HT was always ranked higher overall.

Participants found tasks 4, 5 and 6 more difficult than 1, 2 and 3; we wanted to explore if the task difficulty might affect the participants’ rating of MS Word regardless of the scenario, i.e. the translation modality used. However, when the scores are compared for MS Word, the JA, DE, ES groups do not rate the application significantly differently because of the type of tasks. However, when the EN language was analysed using a Wilcoxon signed rank test, the tasks have a significant effect for the EN group (Z =  − 2.63, *p* = 0.009) when rating MS Word. Eleven participants were less satisfied with MS Word in tasks 1, 2, 3; two were more satisfied with MS Word in tasks 4, 5, 6, and 5 had no preference. This shows that even if the difficulty of the tasks played a role in how participants rated MS Word for the JA, DE, ES groups, the language played a more significant role.

### Retrospective think aloud

All interviews were transcribed and coded using NVivo. However, due to the length of the present article, a summary of themes and findings is presented here.

#### Different scenarios

Possibly the most surprising comment from the RTA was that the JA, DE, ES groups did not notice, with only a few exceptions in the JA and DE groups, that the MS Word application was different after returning from the pause. This might be expected in the ES group due to quality of MT in this language pair (although the MT Scenario had two menus with the same name, hence Design and Layout were translated with the same noun, *Diseño*), but somewhat surprising for the DE and JA groups. We speculate that the participants were concentrating on the completion of the tasks, and since they were not informed that there was a change in the application, they assumed they were working with the same application. However, this also refers to the high quality of the MT engine used by Microsoft.

#### Language in the Word application

Overall, when asked about the quality of the language, the participants stated that it was “good”, “okay”, “alright”, “pretty clear”, “correct”, “easy” in both scenarios. However, target language participants did report on words that were wrong, incorrect or confusing (as per Table 1) in the MT scenario, and these terms created difficulties when completing the tasks. To overcome these difficulties, they resorted to their previous experience, checked the context, checked the visual icons, or they back-translated the term (into English), as in Lim and Fussell (2017) and Pituxcoosuvarn et al. (2018).

Some participants also commented on the overall MS Word design, they found that certain options were placed incorrectly, not in the “logical” place, or they were difficult to find (i.e. Word Options in the File menu), that the naming convention was at times obscure (i.e. Learning tools), formal or too technical, or that the application was not user-friendly in comparison to others, such as Google Docs.

#### Instructions and questionnaires

Although, participants stated that instructions in their own language were clear overall, some preferred step by step instructions (as in a User Guide) rather than an overall description to achieve a goal (as set in this experiment). Participants also mentioned that some terms in the instructions posed difficulties such as the term *dialog box* or *menu* (in all languages) which points to the experience in using word-processors, but also to a different profile of users: a younger generation might be less familiar with this terminology. Others mentioned that the instructions for the first three tasks were clearer than those for the second three tasks, but this could also be related to the difficulty of the task itself.

#### Tasks

Most participants reported that they found the first three tasks easier to complete than the second three tasks, and they found task 4 particularly difficult as has been observed in the quantitative analysis. As per the self-reported questionnaire and the results, the JA group reported having more difficulties with certain tasks than the other groups, and less experience with those tasks and MS Word in general. They also had more difficulties with the English language (see Sect. 3.6), so it was harder for them to explain their experience during the RTA.

#### Experience

The participants often referred to their previous experience when explaining their performance, i.e. familiarity or frequency of use. Further, participants who had experience and worked quickly through the tasks, explained that they were not really “reading” the menus, options or buttons. Those that did not have experience refer to the use of images and emergent menus (those help menus that appear when users hover over an option) to help them find the solutions.

## Conclusions

Our overarching question was *What is the impact of translation modality on the user experience?*, and we articulated this through four research questions. The results show that the variable Effectiveness is not significantly different according to the translation modality although for the DE and JA groups the completion percentages are higher for the HT scenario. However, the variables Efficiency and Satisfaction are significantly different according to the translation modality, and this is especially true for the JA group, with the least experience, when working in the MT scenario. Surprisingly, the ES group is more effective and efficient in the MT scenario, although participants reported a higher satisfaction in the HT scenario. All participants noticed words that were wrong, strange, or confusing in the MT scenario, and they compensated this lack of understanding with context, visuals or back translation, and this is what they remembered when rating Satisfaction in both scenarios. The Satisfaction is also lower for more difficult tasks, and this might indicate that the less familiar we are with an application—the lower the experience—the more we need “high quality” translation to help us navigate our way around that application, and MT might have a negative impact on the user experience. Nevertheless, if MT is to be used, it seems that applications need to support this translation modality by having more images or contextual help to aid users.

When participants are asked specifically about the language in MS Word, they report being more satisfied in the HT scenario, even if it went unnoticed by most participants that the MS Word setup had been changed during the experiment (from HT to MT; and from MT to HT depending on the group order). This perceived value is a key factor when customer experience and retention need to be considered by software companies when implementing MT solutions, even if it is unknown to the users that machine-translated content is used.

Another aspect to consider is that when participants complete fewer tasks (Effectiveness) they tend to rate their Satisfaction lower because they feel that either themselves, the instructions, or the language is inadequate, and this was significant for the English group when rating the language in the MS Word application. Their satisfaction was lower after doing the most difficult tasks, even if the mode of production for the language (the translation modality) had not changed.

Furthermore, participants’ experience (as may be expected) plays a significant role, although moderate, in the Effectiveness, Efficiency and Satisfaction scores. This experience also means that the participants who are very familiar with a task in an application hardly need to “read” the text to know what to do. The task becomes automated for them. Therefore, users who are new to a task or an application need higher levels of clarity and accuracy in the language used, and the use of MT might compromise this for novel users, alternatively the users need more contextual or visual information to find their way through the application.

Finally, when looking at data from the eye-tracker, participants experienced a higher cognitive load overall when working in the translated rather than the original English version; if we look at the Fixation Mean value, the EN group shows significant lower values when looking at the instructions and when working with Word. If we look at the Fixation mean, there are no significant differences between scenarios (HT or MT), but only between the first and the second set of tasks. This highlights the fact that the instructions were originally written in English and that the application is designed primarily in English, and then translated. It would be interesting to further test this finding with other applications and their translated versions.

Would this have been different if participants were using a system translated using NMT? As we can see from the literature when comparing both paradigms (Bentivogli et al. 2016; Castilho et al. 2017; Castilho and Guerberof Arenas 2018; Toral et al. 2018; Daems and Macken 2019; Läubli et al. 2019) improvements in quality have been observed when moving from SMT to NMT systems, but the effect this improvement has on end-users, if any, has yet to be defined clearly. When reading within a software application (with a focus on completing a task), as in this experiment, the important factor appears to be key words, i.e. accuracy, not necessarily the fluency of the text, which is where NMT performs better. Therefore, if raw NMT output is used (especially if compared to a highly customized SMT system as in this experiment), the results might be similar because users might also notice or be confused by incorrect or unclear terms and report lower satisfaction scores. This remains to be tested.

## Appendix 1: Instructions

Warm up Task: change font. You will now see a paragraph in a Word document. Please, do not read the text. You just need to select the text by clicking three times on the text (do not drag the mouse to select), and then change the existing font to a different one. Once you have completed this task, press F10 to move on to the next one.
TASK 1: the digital pen. At the end of the document, create a circle using a digital pen from the menu that contains drawing options. Do not worry if the shape of the circle is not perfect. Make sure the size of the pen is 0.5 mm in thickness. Make sure that the pen colour is set to dark blue. Once you have completed this task, press F10 to move on to the next one.
TASK 2: the paragraph. Using the dialog box for paragraph options, specify the indentation for the given paragraph. Type 2 cm for the left indentation, and 1 cm for the right indentation. In the spacing option, type 12 points to set the spacing after the paragraph. Once you have completed this task, press F10 to move on to the next one.
TASK 3: review document. Using the appropriate menu to review documents, select the option to verify grammar and spelling. Correct any errors only if specified by Word. Do not correct errors that you might deem necessary. Once you have completed this task, press F10 to move on to the next one.
TASK 4: Word options. Access the Word options dialog box through the appropriate menu. On the left panel, select the option that allows you to change how Word corrects and formats the text. In the dialog box, search for the checkbox that allows you to spot words that are frequently confused for others and select this option. Once you have completed this task, press F10 to move on to the next one.
TASK 5: breaks. In the appropriate menu, search for the option that allows you to insert a section break in the document. Select the option to insert a section break, not a page break, to start a section in the next page after the paragraph. Once you have completed this task, press F10 to move on to the next one.
TASK 6: the learning tools. In the appropriate menu, select the Learning Tools. Make sure the page is set to a black background with white letters. Once you have completed this task, press F10 to move on to the next one.

## Appendix 2

See Tables

**Table 2:** Effectiveness according to tasks, scenario and language

Tasks/scenario/language			N	Mean (%)	SD (%)
1, 2, 3	HT	JA	12	82.64	9.70
		DE	9	92.59	10.58
		ES	10	82.50	23.39
	MT	JA	10	74.17	27.06
		DE	10	93.33	12.30
		ES	10	96.67	5.83
	EN	EN	18	93.98	12.06
4, 5, 6	HT	JA	10	46.67	26.99
		DE	10	73.33	27.44
		ES	10	55.00	33.38
	MT	JA	12	40.28	29.05
		DE	9	50.00	42.49
		ES	10	61.67	36.68
	EN	EN	18	62.96	34.09

^2^,

**Table 3:** Efficiency according to tasks, scenario and language

Task/scenario/language			N	Mean	SD
1, 2, 3	HT	JA	12	31.92	13.89
		DE	9	43.22	18.74
		ES	10	36.81	17.29
	MT	JA	10	21.13	8.47
		DE	10	38.33	14.67
		ES	10	35.94	12.09
	EN	EN	18	48.75	19.27
4, 5, 6	HT	JA	10	11.88	9.64
		DE	10	24.01	18.71
		ES	10	10.6	8.26
	MT	JA	12	9.11	8.08
		DE	9	12.16	11.08
		ES	10	15.31	10.58
	EN	EN	18	21.63	19.94

^3^,

**Table 4:** Mean fixation duration in AOIs in seconds

Task/scenario/language			N	Instructions		MS Word	
				Mean	SD	Mean	SD
1, 2, 3	HT	JA	10	65.18	29.16	139.84	70.57
		DE	9	77.11	21.49	115.43	50.74
		ES	10	56.72	17.03	139.62	94.86
	MT	JA	8	75.33	29.76	173.42	92.75
		DE	10	73.56	27.66	148.83	92.27
		ES	10	69.64	20.17	150.87	55.23
	EN	EN	18	54.13	22.16	88.21	44.33
4, 5, 6	HT	JA	8	84.18	37.03	190.21	187.73
		DE	10	105.94	41.75	213.44	111.06
		ES	10	110.34	39.14	339.5	131.07
	MT	JA	10	117.90	51.85	244.71	100.71
		DE	9	94.43	43.90	233.27	123.64
		ES	10	93.39	31.91	243.03*	77.38
	EN	EN	18	78.07	55.02	223.21	215.16

^4^ and

**Table 5:** Satisfaction according to tasks, scenario and language

Task/scenario/language			N	Mean	SD
1, 2, 3	HT	JA	12	3.42	1.33
		DE	9	1.75	0.59
		ES	10	1.4	0.5
	MT	JA	10	3.45	1.06
		DE	10	2.05	0.82
		ES	10	2.13	1.27
	EN	EN	18	2.14	1.02
4, 5, 6	HT	JA	10	3.55	1.21
		DE	10	2.8	1.36
		ES	10	2.98	1.29
	MT	JA	12	4.65	1.25
		DE	9	3.22	1.14
		ES	10	2.55	1.3
	EN	EN	18	3.24	1.15

^5^.

## Appendix 3: Eye-tracking complementary data

### Fixation duration

Fixation duration looks at the duration of fixations in seconds within an AOI.

**Table:**

Task/scenario/language				Instructions		MS Word	
			N	Mean	SD	Mean	SD
1, 2, 3	HT	JA	10	65.18	29.16	139.84	70.57
		DE	9	77.11	21.49	115.43	50.74
		ES	10	56.72	17.03	139.62	94.86
	MT	JA	8	75.33	29.76	173.42	92.75
		DE	10	73.56	27.66	148.83	92.27
		ES	10	69.64	20.17	150.87	55.23
	EN	EN	18	54.13	22.16	88.21	44.33
4, 5, 6	HT	JA	8	84.18	37.03	190.21	187.73
		DE	10	105.94	41.75	213.44	111.06
		ES	10	110.34	39.14	339.50	131.07
	MT	JA	10	117.90	51.85	244.71	100.71
		DE	9	94.43	43.90	233.27	123.64
		ES	10	93.39	31.91	243.03	77.38
	EN	EN	18	78.07	55.02	223.21	215.16

### Fixation count

Fixation count looks at the total number of fixations within an AOI.

**Table:**

Task/scenario/language			N	Instructions		MS Word	
				Mean	SD	Mean	SD
1, 2, 3	HT	JA	10	343.50	101.47	631.40	300.09
		DE	9	393.22	100.83	413.89	210.46
		ES	10	305.70	87.81	489.20	350.66
	MT	JA	8	402.00	147.46	731.25	336.08
		DE	10	385.50	111.79	563.40	351.26
		ES	10	354.00	62.96	578.80	176.22
	EN	EN	18	316.44	101.42	404.06	179.45
4, 5, 6	HT	JA	8	448.63	226.25	939.63	963.72
		DE	10	545.90	182.18	962.50	491.03
		ES	10	550.80	148.98	1510.2	659.94
	MT	JA	10	585.80	214.14	1175.6	439.54
		DE	9	471.22	183.26	1000.2	498.42
		ES	10	489.30	145.74	1055.7	318.04
	EN	EN	18	452.06	287.22	1142.6	918.70

### Visit duration

This gives the duration of each individual visit within an AOI in seconds, in other words, how long participants spent in that AOI before moving to another.

**Table:**

Task/scenario/language			N	Instructions		MS Word	
				Mean	SD	Mean	SD
1, 2, 3	HT	JA	10	88.82	29.52	193.77	90.06
		DE	9	96.78	27.17	141.50	68.09
		ES	10	73.25	20.50	170.91	115.50
	MT	JA	8	101.28	42.97	227.32	111.51
		DE	10	92.74	27.18	180.86	99.89
		ES	10	87.94	19.37	188.80	61.82
	EN	EN	18	77.52	29.48	133.03	67.44
4, 5, 6	HT	JA	8	118.60	68.82	264.16	271.72
		DE	10	135.03	41.82	269.16	133.69
		ES	10	143.02	45.47	447.46	221.10
	MT	JA	10	156.23	57.83	341.29	116.66
		DE	9	117.17	49.85	285.38	147.25
		ES	10	121.12	37.03	314.06	87.62
	EN	EN	18	112.38	71.21	345.08	275.48

### Visit count

This measures the number of visits between the first fixation on the active AOI and the end of the last fixation within the same active AOI where there have been no fixations outside the AOI.

**Table:**

Task/scenario/language			N	Instructions		MS Word	
				Mean	SD	Mean	SD
1, 2, 3	HT	JA	10	38.20	10.40	38.20	11.23
		DE	9	24.67	9.64	25.56	10.06
		ES	10	27.10	10.27	28.00	10.39
	MT	JA	8	39.88	15.18	40.13	13.07
		DE	10	30.90	8.94	32.40	11.13
		ES	10	28.90	9.68	32.30	11.47
	EN	EN	18	32.83	11.01	33.06	11.41
4, 5, 6	HT	JA	8	40.13	21.96	41.63	25.07
		DE	10	34.90	12.37	40.70	16.89
		ES	10	40.10	15.54	50.40	18.14
	MT	JA	10	49.50	18.06	64.80	31.04
		DE	9	28.00	13.75	35.67	16.92
		ES	10	38.10	12.78	50.30	38.30
	EN	EN	18	46.11	28.29	48.11	24.56

## Footnotes

1 https://www.statista.com/forecasts/963597/software-revenue-in-the-world.

2 https://www.nimdzi.com/software-localization-vertical-report-full-report.

3 https://www.statista.com/statistics/257656/size-of-the-global-language-services-market/.

4 https://euatc.org/wp-content/uploads/2019/11/2019-Language-Industry-Survey-Report.pdf.

5 https://euatc.org/wp-content/uploads/2019/11/2019-Language-Industry-Survey-Report.pdf.

6 This is unsurprising since MT engines produce variable quality for different languages.

7 International Organization for Standardization. 2002. ISO/TR 16,982: Ergonomics of human-system interaction – Usability methods supporting human centered design. Available on-line http://www.iso.org/iso/iso_catalogue/catalogue_tc/catalogue_detail.htm?csnumber=31176 (last accessed December 16th 2019).

8 A translation memory is a repository of previously translated segments.

9 https://hub.microsofttranslator.com/.
